# Differential methylation at the *RELN* gene promoter in temporal cortex from autistic and typically developing post-puberal subjects

**DOI:** 10.1186/s11689-016-9151-z

**Published:** 2016-04-29

**Authors:** Carla Lintas, Roberto Sacco, Antonio M. Persico

**Affiliations:** Unit of Child and Adolescent Neuropsychiatry, University Campus Bio-Medico, Rome, Italy; Laboratory of Molecular Psychiatry and Neurogenetics, University Campus Bio-Medico, Rome, Italy; Unit of Child and Adolescent Neuropsychiatry, “Gaetano Martino” University Hospital, University of Messina, via Consolare Valeria 1, I-98125 Messina, Italy; Mafalda Luce Center for Pervasive Developmental Disorders, Milan, Italy

**Keywords:** Autism, DNA methylation, Epigenetics, Post-mortem brains, Reelin

## Abstract

**Background:**

Reelin plays a pivotal role in neurodevelopment and in post-natal synaptic plasticity and has been implicated in the pathogenesis of autism spectrum disorder (ASD). The reelin (*RELN*) gene expression is significantly decreased in ASD, both in the brain and peripherally. Methylation at the *RELN* gene promoter is largely triggered at puberty, and hypermethylation has been found in post-mortem brains of schizophrenic and bipolar patients.

**Methods:**

In this study, we assessed *RELN* gene methylation status in post-mortem temporocortical tissue samples (BA41/42 or 22) of six pairs of post-puberal individuals with ASD and typically developing subjects, matched for sex (male:female, M:F = 5:1), age, and post-mortem interval.

**Results:**

ASD patients display a significantly higher number of methylated CpG islands and heavier methylation in the 5′ region of the *RELN* gene promoter, spanning from −458 to −223 bp, whereas controls have more methylated CpG positions and greater extent of methylation at the 3′ promoter region, spanning from −222 to +1 bp. The most upstream promoter region (−458 to −364 bp) is methylated only in ASD brains, while the most downstream region (−131 to +1 bp) is methylated exclusively in control brains. Within this general framework, three different methylation patterns are discernible, each correlated with different extents of reduction in reelin gene expression among ASD individuals compared to controls.

**Conclusions:**

The methylation pattern is different in ASD and control post-mortem brains. ASD-specific CpG positions, located in the most upstream gene promoter region, may exert a functional role potentially conferring ASD risk by blunting *RELN* gene expression.

**Electronic supplementary material:**

The online version of this article (doi:10.1186/s11689-016-9151-z) contains supplementary material, which is available to authorized users.

## Background

Autism spectrum disorder (ASD) represents a heterogeneous collection of neurodevelopmental conditions characterized by social and communication deficits, accompanied by stereotypic and rigid patterns of behaviors, restricted interests, and unusual sensory processing with onset in early childhood [1]. Neuroanatomical and neuroimaging studies on brains of autistic patients have shown abnormalities stemming from deranged neurodevelopmental processes physiologically occurring during the first and second trimester of pregnancy [2, 3]. During embryogenesis, neuronal and glial precursor cells migrate out of proliferative zones to reach their final destination, where they soon establish intercellular connections. The reelin protein plays a pivotal role during neurodevelopment by acting as a stop signal for migrating neurons in several districts of the central nervous system (CNS), including the neocortex, the cerebellum, and the hindbrain [4–6]. The critical role of reelin in CNS formation has been elucidated by observing profound migration and cytoarchitectonic alterations in *reeler* mice, a loss of function model due to a spontaneous heterozygous deletion of the reelin (*RELN*) gene [7]. Although reelin was initially studied in the context of CNS embryonic development, more recent reports have focused on the role of reelin in the adult brain, particularly in memory processes and in higher cognitive functions [5, 6]. Post-natally, reelin is expressed at high levels primarily in the GABAergic interneurons of the cerebral cortex, hippocampus, and olfactory bulb; perhaps even more relevant to ASD, synaptic strength and plasticity are enhanced by reelin signaling upon binding to apoER2 and VLDLR receptors and subsequent activation of NMDA and AMPA receptors [6, 8–10]. In addition, it exerts a proteolytic activity on extracellular matrix proteins, which is inhibited by organophosphates [11]. Reelin signaling is also required for the development of dendritic spines, which are crucial for signal transmission between neurons. Abnormal shape and reduced numbers of dendritic spines have been found in the reeler mouse and in some subjects affected by autistic disorder, fragile X syndrome, and Rett syndrome [12, 13]. Finally, a significant reduction of dopamine D2 and serotonin 5-HT2A receptors was also reported in the frontal cortex of the reeler mouse as well as in schizophrenic patients [14]. In summary, reelin occupies a pivotal position in the CNS, exerting either directly or indirectly a profound influence on brain structure and function through lifetime. The RELN gene contains at least two alternative isoforms, one with an alternative polyadenylated site and another one with a 6-bp-long microexon [15, 16]. These isoforms are conserved across species and may affect RELN gene expression being located at the 3′ end.

This prominent physiological role immediately links abnormal reelin expression to many neurodevelopmental and psychiatric disorders, including ASD, schizophrenia, bipolar disorder, major depression, and Alzheimer’s disease [17]. Homozygous *RELN* gene mutations are known to produce in humans a rare recessive disease, the Norman-Roberts syndrome, characterized by lissencephaly and cerebellar hypoplasia, with severe mental retardation, abnormal neuromuscular connectivity, and congenital lymphoedema [18]. Less disruptive genetic variation has been found to confer autism risk. Common *RELN* gene polymorphisms yielding lower reelin gene expression both in vitro and in vivo have been found significantly associated with autism in many, though not all, studies [19–21]. Accordingly, post-mortem brain studies have documented reductions in *RELN* gene expression both in the cerebral and cerebellar cortices of ASD individuals compared to controls [22]. Similar reductions have been found measuring reelin plasma levels in vivo [23, 24]. Gene-gene interactions, especially among genes involved in reelin pathway, as well as gene-environment interactions involving various agents and conditions known to enhance autism risk, such as pre-natal immune activation, appear likely [25–29].

In addition to genetic variation, epigenetic control of *RELN* gene expression has also been called into play, initially by studies focused on schizophrenia [30]. The importance of epigenetics in regulating *RELN* gene expression is also supported by our previous results, documenting prominent increases in *RELN* gene promoter methylation in post-mortem temporocortical specimens of post-puberal compared to pre-puberal typically developing individuals [31]. In particular, post-puberal subjects displayed elevated methylation, especially at CpG positions located between −222 and +1 bp, compared to pre-puberal subjects who displayed little or no methylation at all (Additional file 1: Figure S1) [31]. Abnormal methylation patterns at the *RELN* gene promoter have been described in post-mortem brains of schizophrenic and bipolar disorder patients [30, 32, 33]. In addition to the *RELN* gene, other important neurodevelopmental genes, such as UBE3A, GABA receptor genes, and their regulator MeCP2, have also been found epigenetically dysregulated in post-mortem ASD brains [34]. Hence, epigenetic control could play functional roles perhaps even more sizable and widespread than the influence exerted by single functional genetic variants in conferring vulnerability to complex behavioral disorders, such as schizophrenia and autism, or in shaping their clinical course, especially after puberty.

To explore this hypothesis, we have mapped and quantified *RELN* gene promoter methylation assessing in parallel messenger RNA (mRNA) expression levels in the post-mortem temporocortical gray matter (Brodmann area 41/42 or 22) of six post-puberal ASD case-control pairs matched for sex, age, and post-mortem interval (PMI).

## Methods

### Brain tissue samples

Frozen post-mortem brain tissues dissected from the superior temporal gyrus (BA 41/42 or 22, depending on tissue availability) of six post-puberal ASD-control pairs (Table 1) and three ASD pre-puberal subjects were obtained from the NICHD Brain and Tissue Bank for Developmental Disorders at the University of Maryland and the Harvard Brain Tissue Resource Center, through the Autism Brain Net (www.autismbrainnet.org). This neocortical region was chosen because it plays a crucial role in social cognition and hosts well-documented abnormalities in ASD [35]. Largely overlapping post-mortem brain samples have been the object of several previous reports from our group [31, 36–39]. Patients and controls were matched by sex (male:female, M:F = 5:1), age (±2 years) and PMI (Table 1).

**Table 1:** Brain tissue information for ASD patients and controls

Pair no.	Case no.^a^	BA	Diagnosis	Age (years)^b^	Sex	PMI (h)^c^	Cause of death	Intellectual disability	Epilepsy	Other features	Drug therapies at time of death^d^
1	AN11989 (B6677)	41/42	Autism	20	M	16	Cardiac arrest	Yes	Yes	–	Heart medication, Zoloft, clomipramine
2	AN08792 (B5173)	41/42	Autism	30	M	20	Gastrointestinal hemorrhage	Yes	Yes	Large ear lobes	Dilantin, Depakote, Tranxene bid, cisapride, clorazepate, folic acid, oxcarbazepine
3	AN00764 (B5144)	22	Autism	20	M	23.7	Trauma	Yes	No	–	None
4	AN17138 (B6294)	41/42	Autism	16	M	Unknown	Seizure	Unknown	Yes	Inherited 15q duplication	Topamax, Depakote, Allegra, Claritin, NuThera multivitamin
5	AN09730 (B6337)	22	Autism	22	M	25	Seizure	Yes	Yes	Intestinal lymphadenopathy, hypertrophic spleen, recurrent otitis	Lamictal, Zonegran, Neurontin, Abilify, flax seed oil, omega-3, multivitamin
6	AN01570 (B6184)	41/42	Autism	18	F	6.75	Seizure	Unknown	Yes	Focal slowing in the left parieto-occipital area	None
1	AN10833 (B5718)	41/42	Control	22	M	21.5	Unknown	No	No	–	None
2	B4211	41/42	Control	30	M	23	Cardiac arrhythmia	No	No	–	None
3	AN04432 (B3829)	22	Control	22	M	12	Central hepatic laceration	No	No	–	None
4	AN17425 (B6207)	22	Control	16	M	26	Ischemic heart attack	No	No	–	None
5	AN14368 (B6221)	41/42	Control	22	M	24	Unknown	No	No	–	None
6	UMB1541	22	Control	20	F	19	Head injuries	No	No	–	None

*BA* Brodmann area, *PMI* post-mortem interval

^a^Autism Tissue Program identifier

^b^Mean age (±SD) for the autism group = 21.0±2.9, for controls = 22.0±1.8; *t* = −0.368, df = 10, *p* = 0.721

^c^Mean PMI (±SD) for the autism group = 18.2+3.2, for controls = 20.9±2.0, *t* = −0.707, df = 9, *p* = 0.497

^d^Pharmacological therapy from the last available report, dating back to less than a year prior to death

### Bisulfite treatment

DNA was recovered by phenol/chloroform extraction and ethanol precipitation, following brain tissue digestion with proteinase K at 55 °C overnight. One microgram of genomic DNA dissolved in 20 μl of distilled water was denatured in 0.3 M NaOH for 15 min at 37 °C and treated with a freshly prepared urea-sodium bisulfite solution (5.36 and 3.44 M, respectively, at pH 5.0), in the presence of 0.5 mM hydroquinone. Samples were mixed and incubated for 6 cycles, each encompassing 15 min at 55 °C and 30 s at 95 °C, followed by 14 h at 55 °C. DNA was then purified using a desalting column (Wizard Clean Up, Promega), according to the manufacturer’s protocol. Desulfonation and ethanol precipitation were then carried out, as described in [40]. To minimize interindividual variability due to experimental conditions, all samples, positive control and negative control, were processed simultaneously. An in vitro methylated DNA, obtained using the CpG methyltransferase M.SssI (New England Biolabs, Ipswich, MA), was used as a positive control for bisulfate conversion. As a negative control, tissue from UMB1185 was used. This tissue derives from a control pre-puberal subject, which was previously shown to be entirely unmethylated, and thus, it demonstrates a complete C-to-T conversion during bisulfite treatment [31].

Post-mortem confirmation of consent was obtained from next of kin for use of donor brain tissue and publication of individual details presented in this manuscript. Consent forms are held by the NICHD Brain and Tissue Bank for Developmental Disorders at the University of Maryland and the Harvard Brain Tissue Resource Center.

### Reelin promoter amplification by nested PCR, DNA cloning, and sequencing

Bisulfite-converted genomic DNA was amplified by nested PCR. The *RELN* gene region spanning from −516 to +344 bp (+1 is the transcription start site; Genbank acc. n. AC002067) was divided into three amplicons, as follows: amplicon 1 outer primers, −516 (forward), 5′-GGAAAAATAGGGTATATTG-3′, and +115 (reverse), 5′-CACATTCAATTTTAAAAAC-3′; inner primers, −495 (forward), 5′-GTTAAAGGGGTTGGTTTTT-3′, and −132 (reverse), 5′-ACCAAACCTAAAAAAAC-3′; amplicon 2 outer primers, −148 (forward), 5′-GTTTTTTTAGGTTTGGT-3′, and +200 (reverse), 5′-CTAAAAAAAAAATCTACC-3′; nesting reverse primer, +115, 5′-CACATTCAATTTTAAAAAC-3′; and amplicon 3 outer primers, −42 (forward), 5′-GGTTTAAAGTAATTTTGGGAGT-3′, and +344 (reverse), CAATATACAAAAAAATAAACACC-3′; nesting forward primer, +97, 5′-GTTTTTAAAATTGAATGAG-3′. First- and second-round PCRs were performed in a total volume of 25 μl including 10 mM Tris-HCl (pH 8.3), 50 mM KCl, 1.5 mM MgCl_2_, 0.8 mM dNTPs, 1.5 U of *TaKaRa Taq*™ Hot Start Version (TaKaRa, Shiga, Japan), and 0.4 μM of each primer (Invitrogen, Carlsbad, CA). Approximately 30 ng of bisulfite-converted genomic DNA was used as a template for the first PCR; 4 μl of a 1:20 dilution of the first PCR product was used as a template for the second PCR. PCR cycling conditions for the first two amplicons were 95 °C for 5 min; 35 cycles at 95 °C for 30 s, 43 °C for 30 s, and 72 °C for 30 s; and 72 °C for 10 min. PCR conditions for the third amplicon differ only in the annealing temperature of the first- and the second-round PCRs which were 53 and 50 °C, respectively. PCR products were analyzed by electrophoresis on a 1.5 % agarose gel, stained with ethidium bromide and visualized under UV light. PCR products were then cloned into the pCR2.1-TOPO cloning vector (Invitrogen, Carlsbad, CA), using the TOPO TA reagents according to the manufacturer’s protocol. DNA sequencing was performed on 17–21 clones per each individual, using a CEQ8000 DNA sequencer (Beckman-Coulter, Fullerton, CA).

### Fragment analysis for GGC genotyping

The GGC repeat was PCR amplified using primers SGR1 5′-CGGCGTCTCCAAAACTGAAT-3′ and with the FAM-labeled SGR2 5′-AACAGCGCTAGGAGGAAAGT-3′. PCR was performed in a total volume of 20 μl using 100 ng of genomic DNA, 10 mM Tris-HCl (pH 8.3), 50 mM KCl, 1.5 mM MgCl_2_, 0.2 mM dNTPs (dATP, dTTP, and dCTP), 0.15 mM dGTP and 0.05 mM deaza-dGTP (Roche Diagnostics GmbH, Mannheim, Germany), Taq Gold polymerase (Applied Biosystems, Foster City, CA, USA), 0.4 μM each primer (Invitrogen, Carlsbad, CA), and 10 % DMSO. Each cycle consists in a denaturation step at 94 °C for 1 min, an annealing step at 65 °C for 1 min, and a polymerization step at 72 °C for 2 min. After PCR, DNA typing was performed by capillary electrophoresis using the ABI 3500 XL Genetic Analyzer (Applied Biosystems, Foster City, CA, USA). The LIZ-labeled ladder (GeneScan 500 LIZ) was used for sizing determinations of the amplified fragments, and the number of fragment repeats was based by comparison with the allelic ladder.

### Real-time PCR

Total RNA was extracted from temporocortical brain tissue immediately adjacent to the tissue specimen used for genomic DNA extraction, using the TRIzol method (Invitrogen, Carlsbad, CA). RNA quality was checked using a Bioanalyzer (Agilent, Santa Clara, CA). RNA (1 μg) was reverse transcribed using the QuantiTect Reverse Trascription kit (Qiagen, Hilden, Germany). *RELN* complementary DNA (cDNA) levels were measured using an ABI PRISM 7900HT Real-Time PCR system using a standard amplification protocol. Total RELN cDNA was measured using a standard SYBR Green-based comparative protocol (2^−ΔΔCt^) [41]. We also measured the proportion of the alternative polyadenylated transcript (polyA) and of the alternatively spliced microexon applying the same delta subtractive Ct methodology described by Ovadia and Shifman [16]. The human CHL1 gene was measured in parallel and used as a normalizer. cDNA dilutions (1:10) were used to quantify all transcripts. All samples were reverse transcribed in duplicate, and cDNA was run in quadruplicate to allow assessment of sample homogeneity and technical variability. Primer sequences were the same as used by Ovadia and Shifman [16] with the exception of the primer set for the microexon for which the following new primers were designed: 5′-GTGGAGGTCGTCCTAGTAAG-3′ forward primer and 5′-TTGATTCTTCATGGGTATCGCC-3′ reverse primer.

The specificity of the PCR product was checked by running a melting curve at the end of each experiment. The efficiency of the PCR amplification was assessed by serial dilutions for each primer set (>0.95 for all sets). Little variability was observed among controls and ASD samples (Additional file 2: Figure S2).

### In silico analysis

The biostatistical softwares Alibaba 2, Match, and P-Match were used to predict the presence of putative transcription factor binding sites in the *RELN* gene promoter (http://www.gene-regulation.com/pub/programs.html).

### Statistical analysis

Methylation was evaluated applying two different indices: (a) “percent of methylation” index (number of methylated clones/total number of clones at each CpG position), to quantify the extent of methylation at each CpG position, and (b) “extent of methylation” index (number of methylated CpG positions in a given individual/total number of CpG positions along the reelin promoter) to study the overall distribution of methylated CpGs along the RELN gene promoter. These two indices essentially reflect the intensity and the extension of methylation along the RELN gene promoter, respectively. A position was given methylated when it was found methylated in at least two clones from the same individual. Considering the small sample size of available brain specimens, non-parametric tests Wilcoxon test for paired data and two-tail probability thresholds were employed to compare RELN gene promoter methylation and expression in ASD vs typically developing controls. Correlation between RELN gene expression levels (expressed as ΔCt) and methylation indices was assessed applying the Spearman correlation index. Post-mortem confirmation of consent was obtained from next of kin for use of donor brain tissue and publication of individual details presented in this manuscript. Consent forms are held by the NICHD Brain and Tissue Bank for Developmental Disorders at the University of Maryland and the Harvard Brain Tissue Resource Center. This study was approved by the I.R.B. of University Campus Bio-Medico (23.06), and it is in compliance with the Helsinki Declaration.

## Results

### Differential methylation at the RELN gene promoter between ASD and control brains

**Fig. 1:**
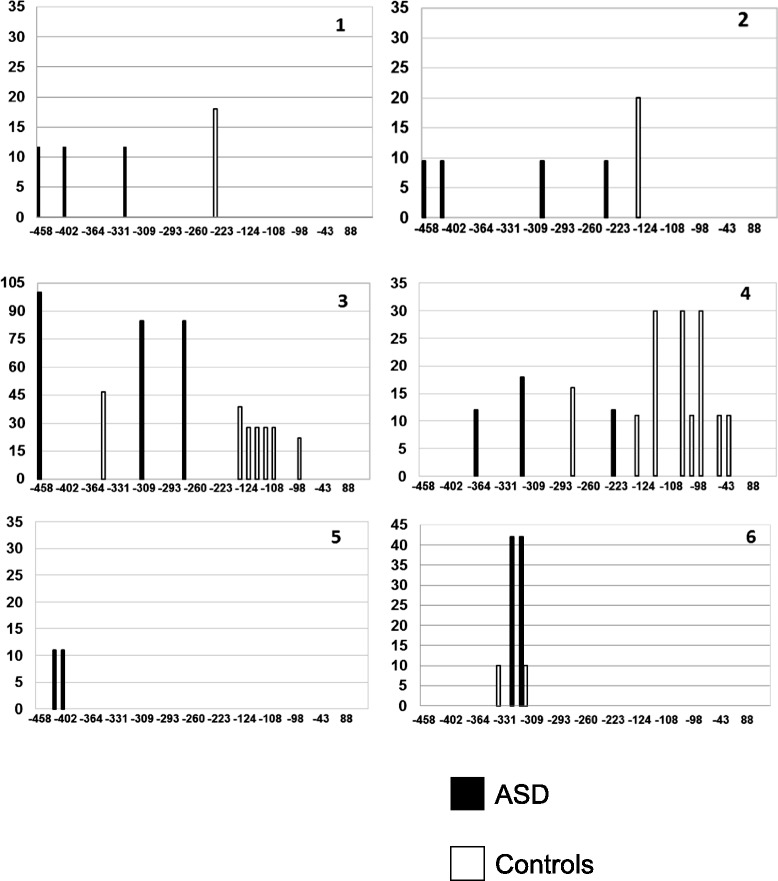
Position of methylated CpGs and percent of methylation at the *RELN* gene promoter in six post-puberal ASD brains (*black bars*) and their matched controls (*white bars*)

**Fig. 2:**
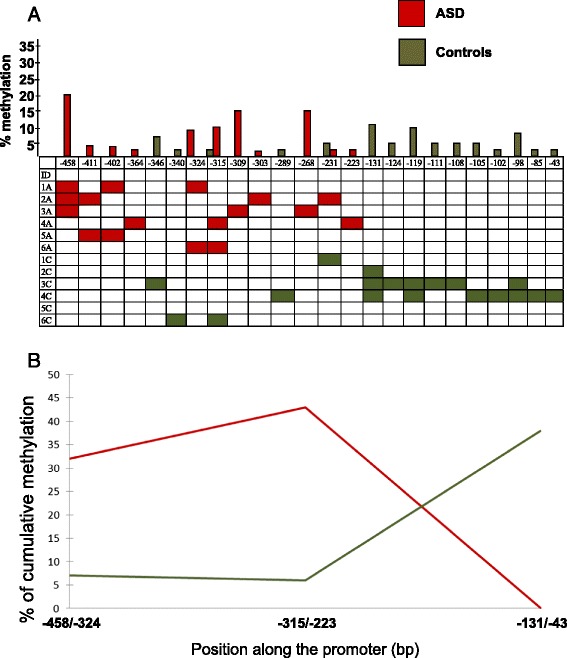
**a** Mean percentage of methylation at each methylated CpG position (*top*) and presence of methylation in the six ASD (*red*) and six control (*green*) temporocortical specimens from post-puberal individuals (pairs n.1–6 in Table 1). **b** Cumulative percentage of methylation in the three regions of the RELN gene promoter. Region 1, from −458 to −324; region 2, from −315 to −223; and region 3, from −131 to −43

ASD and control brains collectively display methylation at 24 different CpG positions distributed along the *RELN* gene promoter between −458 and −43 bp (Figs. 1 and 2). Percentage of methylation and number of methylated CpG positions display broad interindividual variability. The mean percentage of methylation at each CpG position and the mean number of methylated CpGs along the entire *RELN* gene promoter do not differ significantly between ASD and control brains (two-tail *p* = 0.34 and *p* = 0.46, respectively) (Table 2). However, the distribution of methylated CpG positions across the *RELN* gene promoter is different between ASD and control brains. As shown in Fig. 2, ASD brains carry significantly more numerous and more heavily methylated CpG positions in the 5′ portion of the *RELN* gene promoter, spanning from −458 to −223 bp, reaching two-tail *p* values of 0.04 for both indices (Table 2). On the contrary, the 3′ promoter region, spanning from −222 to +1 bp, displays a methylation pattern exclusively restricted to controls (Fig. 2), despite not reaching a significant two-tail *p* value for both methylation indices due to small sample size and slightly lower methylation intensity (Table 2).

**Table 2:** Summary statistics of *RELN* gene promoter methylation indices

	Promoter region	ASD	Controls	*p* value
Mean percent of methylation^a^	5′ region (−458 to −223 bp)	29.55 ± 13.09	5.70 ± 2.90	0.04*
	3′ region (−222 to +1 bp)	0	10.83 ± 5.04	0.10
	Total region (−458 to +1 bp)	29.55 ± 13.09	16.00 ± 4.21	0.34
Mean extent of methylation^b^	5′ region (−458 to −223 bp)	0.16 ± 0.01	0.04 ± 0.01	0.04*
	3′ region (−222 to +1 bp)	0	0.21 ± 0.10	0.10
	Total region (−458 to +1 bp)	0.09 ± 0.01	0.11 ± 0.04	0.46

Data are expressed as means ± standard error of the mean. Two-tail *p* values were obtained using non-parametric Wilcoxon tests*p < 0.05

^a^(mean number of methylated clones at each CpG position/total number of clones) × 100

^b^(mean number of methylated CpG positions in a region/the total number of methylated CpG positions in that region) × 100

**Fig. 3:**
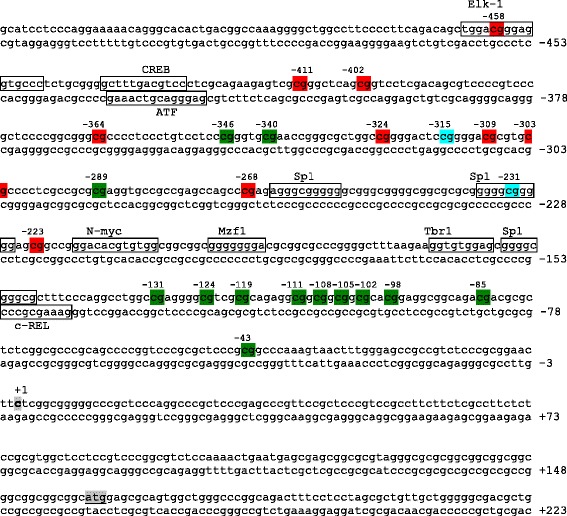
Methylation status at the *RELN* gene promoter in case-control pairs n.1–6. CpGs uniquely methylated in post-puberal ASD or control brains are highlighted in *red* and in *green*, respectively, while CpGs methylated in both are shown in *light blue*. The trascription start site and translation start site are boxed in *gray*. Response elements with high affinity (less than three mismatches with respect to the consensus sequence) are boxed in *black*

CpG positions uniquely methylated in ASD brains are all located in the 5′ promoter region and include −458, −411, −402, −364, −324, −309, −303, −268, and −223 (Figs. 2 and 3). Interestingly, CpG position at −458 is methylated in three ASD subjects with 20 % mean extent of methylation (Fig. 2) and is within the putative DNA binding site of the transcription factor ELK-1. Conversely, other CpG positions, mainly located in the 3′ promoter region, are methylated only in controls, including −346, −340, −289, −131, −124, −119, −111, −108, −105, −102, −98, −85, and −43, (Figs. 2 and 3). CpG position at −131 is methylated in three out of six control brains with 12 % mean extent of methylation (Fig. 2).

No or very little methylation at the *RELN* gene promoter was found in ASD pre-puberal subjects demonstrating that methylation is triggered at puberty (Additional file 1: Figure S1). Similarly, one post-puberal ASD individual with a pituitary dysfunction (AN00493) displayed a pre-puberal-like methylation pattern characterized by only one methylated CpG position (33 % methylation at −289 CpG only) and was thus excluded from the study (data not shown).

### RELN gene expression in post-mortem ASD brains and relationship with distinct methylation patterns

**Fig. 4:**
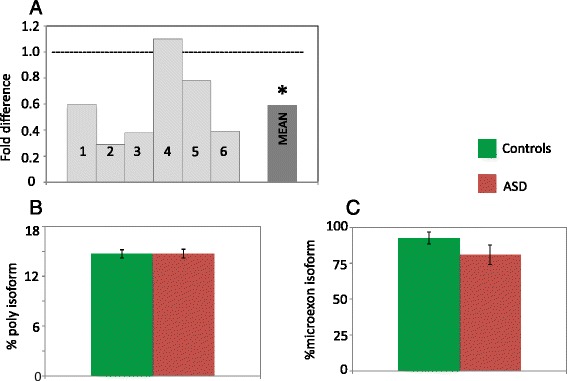
**a** *RELN* gene expression in six ASD post-mortem brains relative to their matched control and mean ASD/control fold difference; the *hyphenated line* corresponds to no case-control difference in mRNA levels. *two-tail *p* < 0.05. **b** Mean proportion of alterative polyadenylation and **c** microexon *RELN* isoforms for controls (*green*) and for ASD (*red*); *error bars* correspond to the standard error of the mean

Autistic brains display a significant reduction in *RELN* total mRNA levels compared to their matched controls (two-tail *p* < 0.05) (Fig. 4a). Instead, no significant difference in the proportion of the RELN alternative polyA and microexon isoforms was detected (Fig. 4b, c). The relationship between gene expression and promoter methylation is not strictly quantitative and linear. In fact, no overall correlation between total *RELN* mRNA levels, expressed as ΔCt, and the two methylation indices was found (*p* > 0.05 for the Spearman correlation index) regardless of diagnostic status. However, three general methylation patterns appear differentially associated with expression levels, based on the ratio between the number of methylated CpGs in each ASD and control pair (compare Fig. 1 with Fig. 4b): (a) pairs n.2, n.3, and n.6 display the heaviest methylated CpGs in ASD compared to controls, corresponding to the most decreased ASD/CON expression ratios, as low as <0.3; (b) pairs n.1 and n.5 have relatively methylated CpGs in ASD compared to little or no methylation in controls, corresponding to even or modestly decreased ASD/CON expression ratios, between 0.6 and 0.8; and (c) pair n.4 shows more and heavier methylated CpGs in controls compared to ASD corresponding to a 1.1-fold change. Within this framework, the extent of CpG methylation seemingly exerts greater influence on gene expression than the overall number of methylated CpG sites (Figs. 1 and 4).

### The RELN GGC triplet repeat does not explain reduced mRNA levels

The presence of “long” GGC repeat alleles does not significantly contribute to these differences in RELN methylation status, as all individuals carry the common 8 and 10 repeat alleles, except for the control subject of pair number 3 (AN04432: genotype 10/12).

## Discussion

The present results provide for the first time a map of *RELN* gene promoter methylation in temporocortical tissue samples from post-puberal ASD and matched control brains, demonstrating differences in the distribution of methylated CpGs, while confirming reductions in *RELN* mRNA levels in ASD brains compared to controls. Our study was restricted to post-mortem brains from post-puberal individuals, since we previously demonstrated a substantial lack of methylation at the *RELN* gene promoter before puberty in control brains [31]. We found the same lack of methylation in ASD pre-puberal brains in this study (Additional file 1: Figure S1). In line with previous data supporting genome-wide epigenetic and transcriptomic abnormalities in ASD brains [37, 42], our results zoom into the *RELN* gene which is relevant, on the one hand, to physiological neurodevelopment and post-natal synaptic management, as well as, on the other hand, to the pathological neurodevelopment underlying several neurodevelopmental disorders, including ASD [17]. Collectively, our results indicate that (a) decreased total *RELN* gene expression is a widespread phenomenon in ASD brains and (b) it seemingly stems from an epigenetic dysregulation resulting in heavier and broader methylation of promoter regions located further upstream those typically methylated in control brains. This epigenetic dysregulation appears independent of autism-associated rare genetic variants, which have an extremely low incidence, and of known functional common variants, including the previously described GGC repeat [19–21]. The proportions of the alternative polyA and microexon *RELN* mRNA isoforms are in line with previously reported data [15, 16], being about 15 % and 80–92 % of total *RELN* mRNA, respectively (Fig. 4b, c).

Several lines of evidence demonstrate that the *RELN* gene promoter is epigenetically controlled, including (a) a switch in the *RELN* gene expression pattern from embryogenesis to post-natal life [43], (b) a significant difference in *RELN* gene promoter methylation and expression levels between pre-puberal and post-puberal brains in typically developing subjects [31], (c) increased *RELN* promoter methylation in post-mortem brains of bipolar disorder and schizophrenic patients [30, 32, 33], and (d) increased *RELN* promoter methylation and downregulation of its expression in rat pups exposed to pre-natal restraint stress or post-natal maternal deprivation [44, 45]. Histone modifications not requiring DNA methylation could be involved in the modulation of gene expression prior to puberty, whereas methylation arises and plays a prominent role in post-puberal brains [31]. However, the present study demonstrates that methylation patterns differ between ASD and control brains. Methylated CpG positions are restricted to the −458- to −223-bp promoter region in post-mortem autistic brains, whereas methylation spans a broader −346- to −43-bp region in matched controls (Fig. 2). ASD and control methylation overlaps between −346 and −231 bp, albeit largely at different CpG sites (Figs. 2 and 3). Two promoter regions display no overlap, namely the upstream −458- to −364-bp region, which is methylated only in ASD brains, and the downstream −131- to −43-bp region, which is methylated exclusively in control brains (Figs. 2 and 3). This difference in the methylation status of the RELN promoter may have important functional consequences on reelin gene expression. For example, CpG position at −458 is uniquely methylated in three out of six autistic patients and localizes inside a putative ELK-1 transcription factor binding site. This factor binds to specific DNA sites containing a central GGA trinucleotide motif [46] and has been found associated with the mitochondria transition pore complex (PTP), a structure involved in both apoptotic and necrotic cell death [47]. In particular, overexpression of ELK-1 in primary neurons decreased cell viability, whereas its knockdown increased cell viability [47]. Hence, the binding of ELK-1 to its response element might be decreased or prevented by specific methylation of this CpG residue, suggesting a putative functional role for this specific position. Alternatively, the chromatin conformation associated with methylation of the −458- to −364-bp region could interfere with ELK-1 binding, even in the absence of methylation specifically at −458 bp. Further work will be necessary to explore these hypotheses. Sex-specific differences in *RELN* expression have been found in several animal model studies. Studies on rats have demonstrated that female progesterone upregulates reelin expression at the periphery [48] whereas the reeler male mouse has a significant higher loss of Purkinje cells in the cerebellum compared to females [49]. In addition, some *RELN* gene SNPs have been associated with schizophrenia and bipolar disorder only in women [50, 51]. We did not notice any sex difference in *RELN* expression and/or methylation pattern in our sample. However, our sample is very small and includes only one female pair.

In addition to differential methylation, most brains of post-puberal autistic subjects display a significant downregulation of reelin gene expression compared to matched controls (Fig. 4b), in line with previous reports [22–24]. Furthermore, we observe three possibly distinct methylation patterns each related to differential degrees of expression blunting in ASD compared to control brains. The relationship between promoter methylation and gene expression levels observed in this study should be viewed as preliminary, but nonetheless, it points toward a functional role of distinct *RELN* gene promoter methylation patterns in differentially modulating *RELN* mRNA expression. Considering the functional relevance of the reelin protein in synaptic management, increased methylation at the 5′ promoter region and downregulation of *RELN* gene expression at puberty could indeed contribute to the worsening of some phenotypic traits observed clinically in many autistic subjects at the onset of adolescence [52]. Well-known clinical features often aggravating or arising soon after puberty include epilepsy and abnormalities of movement, speech, and behavior [52]. In addition to hormonal changes occurring at puberty, also pre-natal and/or early post-natal stress may play a role, since newborn rats whose mothers were exposed to pre-natal restraint stress show increased methylation at the *RELN* gene promoter and, as adults, higher locomotory activity, as well as learning and memory deficits [45]. Similarly, rat pups removed from their mothers during post-natal days 2–15 for 3 h/day show reduced hippocampal *RELN* gene expression and hypermethylation of the *RELN* gene promoter, accompanied by behavioral deficits in auditory startle and grasping reflex, as well as greatly enhanced responses to the hot plate [44]. Collectively, these and the present results suggest that the epigenetic regulation of the *RELN* gene promoter by hormonal and environmental factors may also confer significant vulnerability toward a variety of behavioral disorders, including autism, or influence their developmental trajectory, as does genetic variation at this locus.

The epigenetic regulation of the *RELN* gene promoter is very complex and involves several response elements, bound by specific transcription factors. In general, downregulation of *RELN* gene expression in the brain of schizophrenic and bipolar patients has been linked to the overexpression of DNA methyltransferase 1 (DNMT1) and ten-eleven translocase methylcytosine dioxygenase 1 (TET1) [53]. Furthermore, the neocortex of schizophrenic and bipolar patients displays an increase in DNMT1 binding to the *RELN* gene promoter [54]. This increased binding of DNMT1 positively correlates with increased expression of DNMT1 and with promoter binding of methyl binding domain 2 (MBD2) but not necessarily with enrichment in promoter methylation. This last observation suggests that cytosine methylation may not represent the only mechanism by which DNMT1 modulates gene expression. Indeed, DNMT1 not only binds to methylated CpGs through its methyl binding domain (MBD) but also binds to unmethylated CpGs through its CXXC zinc finger domain [55]. In addition to DNMT1, also MeCP2 binds to the *RELN* gene promoter, modulating its expression. Specifically, an enrichment of 5-hydroxylmethylcytosine relative to 5-methylcytosine seems to mediate an increased binding of MeCP2 to the *RELN* gene promoter in the cerebellar cortex of ASD individuals [56]. Gene expression is thus the result of complex interactions exerted by different biological processes, including DNA methylation/demethylation/hydroxylmethylation, trans-acting chromatin-associated proteins, and non-coding RNAs [17, 57].

The small sample size assessed here, typical of most post-mortem studies, requires caution in interpreting our results as necessarily valid for all patients in such a highly heterogeneous disorder. Independent replications in additional human post-mortem brain samples, investigations employing rodent models, and functional in vitro studies will be needed to clarify the involvement of specific CpG dinucleotides in controlling *RELN* gene expression and their potential role in ASD.

## Conclusions

Methylation patterns in post-mortem neocortical tissue of post-puberal ASD and control individuals are different, with an upstream promoter region methylated specifically in ASD brains, while a downstream region is methylated only in controls. The potential relationship between methylation patterns and *RELN* gene expression observed here is intriguing and deserves further investigation, as do the underlying transcriptional control mechanisms which will now be addressed in follow-up studies. *RELN* gene expression in peripheral lymphocytes has been reported to be very low [58]; however, should methylation patterns at the *RELN* gene promoter display some parallels between brain and leukocytes, it may be possible to assess the clinical correlates of abnormal methylation in autistic individuals at puberty and during adolescence. Finally, it is unclear whether DNA methylation changes are causally inferred, given ASD stress, or represent a core element of the etiology itself. The present results confirm the potential contribution of epigenetics to the definition of biomarker panels for neurodevelopmental disorders [59], although the tissue specificity of methylation marks represents an additional challenge as compared to genetic and biochemical biomarkers.

## Additional files

Additional file 1: Figure S1. A: Position of methylated CpGs and percent of methylation at the RELN gene promoter in three prepuberal ASD brains. B: Brain tissue information for three ASD pre-puberal individuals. ^a^Autism Tissue Program identifier. ^b^Pharmacological therapy from the last available report, dating back to less than a yearprior to death. (PPT 229 kb) Additional file 2: Figure S2. Mean ΔCt value for *RELN* total expression for controls (green) and for ASD brains (red) for the six pairs. Standard errors of the mean are shown. (PPTX 81.5 kb)

## Abbreviations

ASD: autism spectrum disorder
CNS: central nervous system
M:F: male:female
RELN: reelin
