# Supplementary material for: Differential methylation at the RELN gene promoter in temporal cortex from autistic and typically developing post-puberal subjects
Source: J Neurodev Disord. 2016 Apr 29;8:18. doi: 10.1186/s11689-016-9151-z (PMC4850686; doi:10.1186/s11689-016-9151-z)
Supplement: Additional file 1: Figure S1. — A: Position of methylated CpGs and percent of methylation at the RELN gene promoter in three prepuberal ASD brains. B: Brain tissue information for three ASD pre-puberal individuals. aAutism Tissue Program identifier. bPharmacological therapy from the last available report, dating back to less than a yearprior to death. (PPT 229 kb) [file 11689_2016_9151_MOESM1_ESM.ppt]

## Slide 1
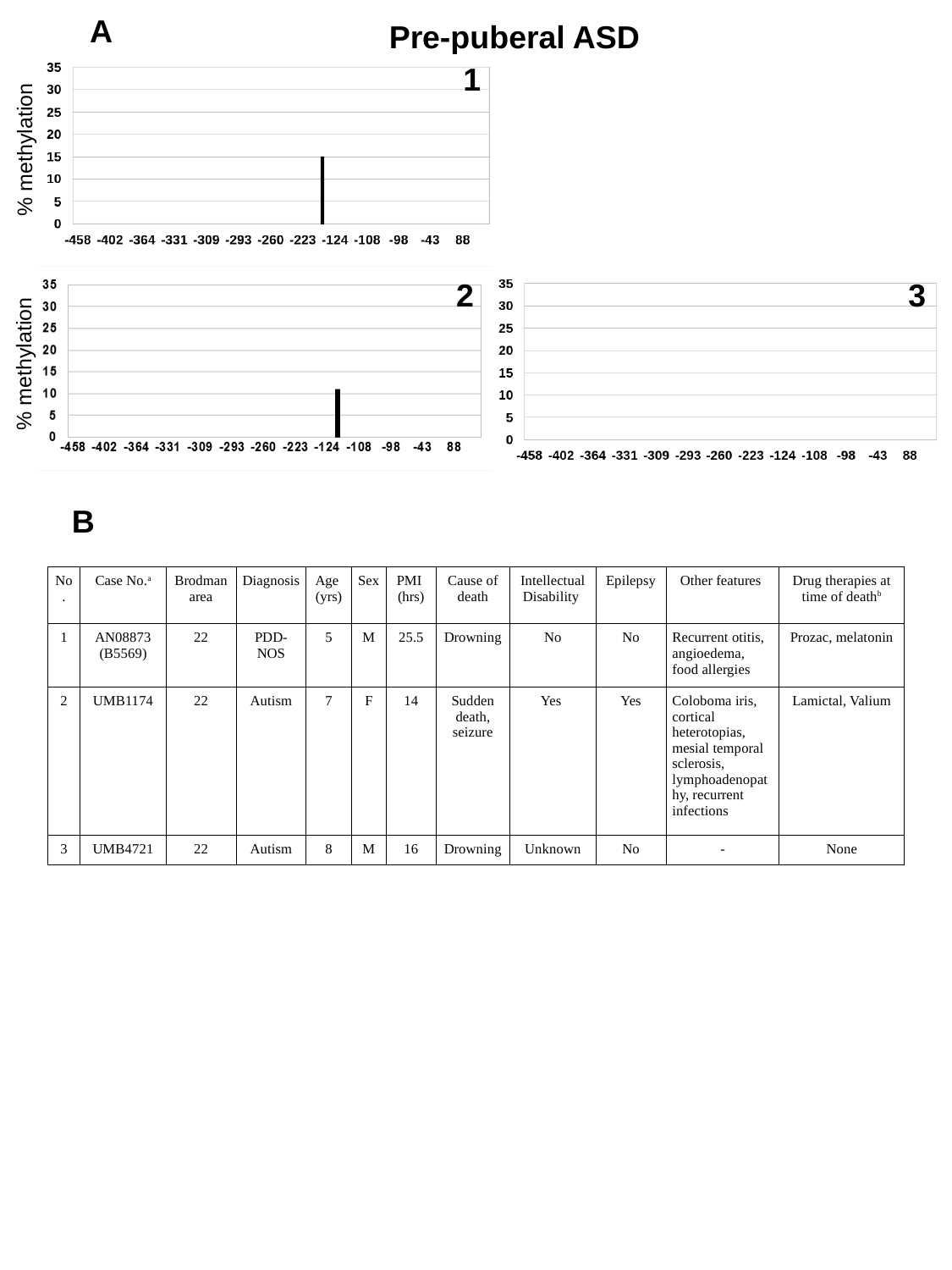

A
 Pre-puberal ASD
1
% methylation
2
3
% methylation
B
| No. | Case No.a | Brodman area | Diagnosis | Age (yrs) | Sex | PMI (hrs) | Cause of death | Intellectual Disability | Epilepsy | Other features | Drug therapies at time of deathb |
| --- | --- | --- | --- | --- | --- | --- | --- | --- | --- | --- | --- |
| 1 | AN08873 (B5569) | 22 | PDD-NOS | 5 | M | 25.5 | Drowning | No | No | Recurrent otitis, angioedema, food allergies | Prozac, melatonin |
| 2 | UMB1174 | 22 | Autism | 7 | F | 14 | Sudden death, seizure | Yes | Yes | Coloboma iris, cortical heterotopias, mesial temporal sclerosis, lymphoadenopathy, recurrent infections | Lamictal, Valium |
| 3 | UMB4721 | 22 | Autism | 8 | M | 16 | Drowning | Unknown | No | - | None |
