# Supplementary figures and images for: Differential methylation at the RELN gene promoter in temporal cortex from autistic and typically developing post-puberal subjects
Source: J Neurodev Disord. 2016 Apr 29;8:18. doi: 10.1186/s11689-016-9151-z (PMC4850686; doi:10.1186/s11689-016-9151-z)

## Slide 1
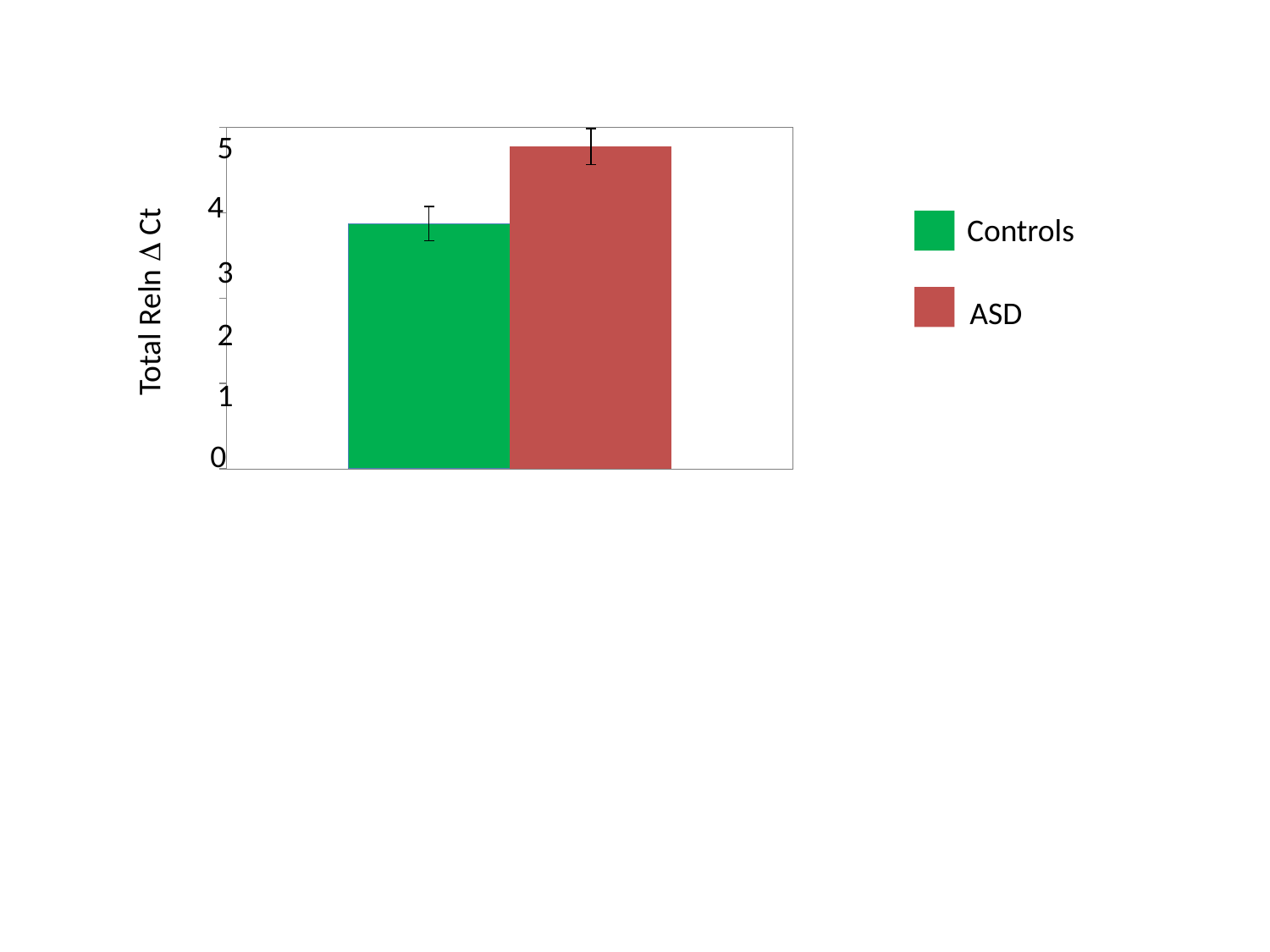

### Chart
| Category | | |
|---|---|---|
| 1 | 2.8750000000000004 | 3.775000000000001 |5
4
Controls
3
Total Reln D Ct
ASD
2
1
0

Supplement: Additional file 2: Figure S2. — Mean ΔCt value for RELN total expression for controls (green) and for ASD brains (red) for the six pairs. Standard errors of the mean are shown. (PPTX 81.5 kb) [file 11689_2016_9151_MOESM2_ESM.pptx]
